# Automatic wild bird repellent system that is based on deep-learning-based wild bird detection and integrated with a laser rotation mechanism

**DOI:** 10.1038/s41598-024-66920-2

**Published:** 2024-07-10

**Authors:** Yu-Chieh Chen, Jing-Fang Chu, Kuang-Wen Hsieh, Tzung-Han Lin, Pei-Zen Chang, Yao-Chuan Tsai

**Affiliations:** 1 Institute of Applied Mechanics, National Taiwan University, Taipei, 106319 Taiwan; 2 Department of Bio-Industrial Mechatronics Engineering, National Chung Hsing University, Taichung, 402202 Taiwan; 3 Smart Sustainable New Agriculture Research Center (SMARTer), Taichung, 402 Taiwan; 4 Graduate Institute of Color and Illumination Technology, National Taiwan University of Science and Technology, Taipei, 106335 Taiwan

**Keywords:** Ecology, Engineering

## Abstract

Wild bird repulsion is critical in agriculture because it helps avoid agricultural food losses and mitigates the risk of avian influenza. Wild birds transmit avian influenza in poultry farms and thus cause large economic losses. In this study, we developed an automatic wild bird repellent system that is based on deep-learning-based wild bird detection and integrated with a laser rotation mechanism. When a wild bird appears at a farm, the proposed system detects the bird’s position in an image captured by its detection unit and then uses a laser beam to repel the bird. The wild bird detection model of the proposed system was optimized for detecting small pixel targets, and trained through a deep learning method by using wild bird images captured at different farms. Various wild bird repulsion experiments were conducted using the proposed system at an outdoor duck farm in Yunlin, Taiwan. The statistical test results of our experimental data indicated that the proposed automatic wild bird repellent system effectively reduced the number of wild birds in the farm. The experimental results indicated that the developed system effectively repelled wild birds, with a high repulsion rate of 40.3% each day.

## Introduction

In recent years, avian influenza has caused substantial economic losses in the poultry farming industry in Taiwan as a result of the death of a large number of birds^1^. According to research reports, the primary transmission routes for avian influenza are direct contact with wild birds and indirect fecal contamination^2^. One of the most effective methods to prevent the spread of avian influenza is to avoid contact between feeding poultry and wild birds. Various methods have been proposed to prevent such contact, and these methods include acoustic deterrents, visual deterrents, chemical repellents, physical techniques, and lethal methods^3^. In terms of acoustic deterrents, ultrasonic frightening devices are not highly effective in repelling wild birds, and scare cannons provide only a temporary solution^4^. To repel wild birds and prevent them from building nests, an improved beam-type ultrasonic device was developed^5^. This device irritates birds and forces them to abandon their nests. In terms of visual deterrents, dead goose effigies have been used in Canada to repel wild birds. However, after a few days, adult birds become accustomed to these effigies, and they thus lose their effectiveness^6^. Some European airports have used trained raptors as a wild bird management strategy^7^. Lasers have also been used in the nighttime dispersal of double-crested cormorants^8^. The efficacy of wild geese repellent by manually operating lasers is very good^9^. However, the total cost is greater than the benefit of preventing damage to rice crops, and most of the total cost is the labor costs. In numerous studies, green lasers have been used to repel wild birds because the majority of wild birds have eyes that contain at least one cone pigment that strongly absorbs green light^10,11^. Thus, the eyes of wild birds are most sensitive to the wavelength of green light. A study was conducted by exposing broilers to four four-minute laser cycles every day. During these cycles, the laser would be projected at random locations in the poultry house. The broilers stimulated by the laser spent more time foraging, particularly during the 1st and 5th weeks. In the 1st, 3rd, and 4th weeks, an increase in broilers activity was found, with a 69.7% increase in the 4th week (*p* < 0.05), and it did not have any effect on the appearance or tibia of the broilers^12,13^. In terms of chemical repellents, foliar chemical sprays combined with plant growth regulators have been used to repel geese from turf grass^14^. In terms of physical techniques, a bird repellent structure comprising a trapezoidal metal plate and U-shaped support fitting has been used at substations to prevent birds from building nests^15^. Exclusion nets have also been highly effective and have been regarded as the most favorable technique for the prevention of wild bird damage to high-value products^16,17^.

In recent years, for automatically repelling wild birds for extended periods while reducing labor costs, attempts have been made to develop an automatic wild bird repellent system. Unmanned aerial vehicles equipped with a wild bird detection system have been used in agricultural farms to reduce the crop damage caused by wild birds^18–20^. An intelligent audio bird repellent system was developed to repel wild birds. This system is capable of identifying the species of an approaching bird on the basis of the bird’s appearance and vocalization through a deep learning network^21^. An automatic bird detection and repellent system based on the Internet of Things was proposed. In this system, a pyroelectric infrared detector is used to detect motion, and a predator sound generator is used to drive birds away from a field^22^. A highly effective automated green laser repellent system was developed to keep wild birds away from a poultry layer farm^23^. A portable, battery-powered, robotic green laser scarecrow was designed for continuously scanning a sweet corn field and using lasers to repel birds. According to the results of split-field experiments, the field scanned by the robotic laser scarecrow had an increased yield because of the absence of birds^24^. However, these laser bird repelling systems of above-mentioned research would not detect wild birds precisely. Most systems are only capable of periodically repelling wild birds within a specific timeframe and limited area. The wild bird repellent system without wild bird detection ability are not as clearly targeted as human to repel wild birds.

There are several methods would be used to detect wild birds, including using radar, lidar, thermal imaging or visible light imaging. Considering cost-effectiveness and practical field usage, visible light imaging is a promising option for wild bird detection. To achieve wild bird detection by visible light images, it is necessary to combine them with object detection technology. Background subtraction has been widely used to detect moving objects in continuous images^25^. Background subtraction combined with shape and motion features has been used to improve the detection accuracy of birds^26^. In recent years, various deep learning techniques, such as faster region-based convolutional neural networks (faster R-CNNs)^27^, You Only Look Once (YOLO)^28^, and Single-Shot MultiBox Detector^29^, have been extensively used for object detection with a bounding box. Mask R-CNN allow the detection model to avoid learning the background noise and to detect targets precisely against complex backgrounds^30^. The detection performance of fast R-CNN, faster R-CNN, region-based fully convolutional networks, single shot detector, deconvolutional single shot detector, R-CNN minus R, you only look once (YOLO), and mask R-CNN were compared. The comparison results found that YOLO has the highest speed performance, while mask R-CNN has the best detection ability^31^. Mask R-CNN and YOLOv3 were used to detect birds, and the results indicated mask R-CNN had superior detection capabilities to YOLOv3^32^.

**Figure 1:**
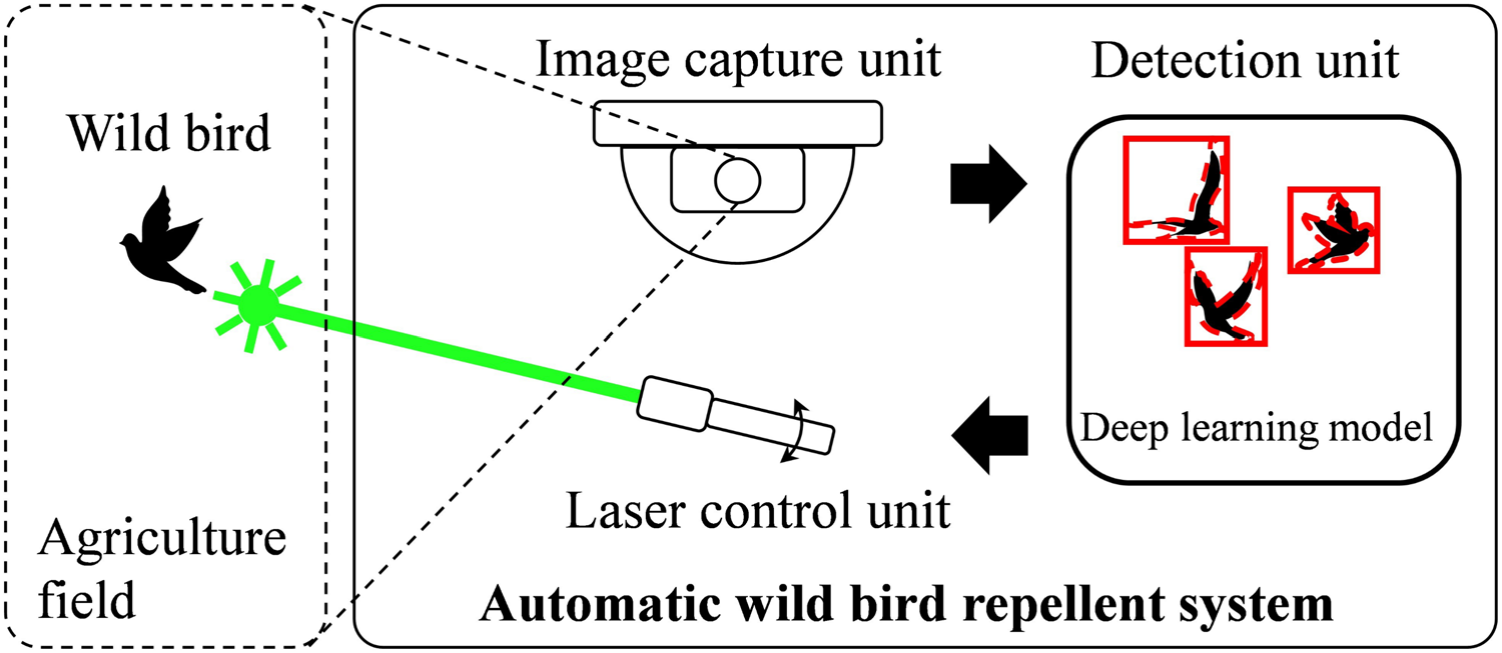
Concept of the proposed automatic wild bird repellent system.

In the present study, we designed and developed an automatic wild bird repellent system that is based on deep-learning-based wild bird detection and integrated with a laser rotation mechanism. As displayed in Fig. 1, the proposed system consists of an image capture unit, a detection unit, and a laser control unit. The image capture unit first captures an image of the agricultural field. The captured image is then sent to the detection unit to detect wild birds by using a deep learning model. If wild birds are detected in the captured image, the laser control unit emits a laser beam to repel the birds. The proposed wild bird detection system hinders wild birds from becoming acclimatized to laser beams, which makes it effective as a wild bird repellent. The effectiveness of the proposed system in repelling wild birds was tested at an outdoor duck farm in Taiwan.

## Materials and methods

### Model selection and image data collection and annotation.

In the proposed system, a wild bird detection model detects the position of a wild bird in an image captured by a camera sensitive to visible light. This model is established and trained using a mask R-CNN deep learning method, and the backbone used in this work is ResNet-101-FPN. Although many object detection techniques are robust and precise, they cannot be used to detect wild birds in outdoor images because wild birds occupy only approximately 20 × 20 pixels in an image with 1920 × 1080 pixels. In addition, background noise and occlusion exacerbate the difficulty of detecting wild birds. Therefore, the mask R-CNN model could be used in embedded systems to detect wild birds. However, the detection performance of the trained model depends on the image dataset used for training. Therefore, appropriate image dataset must be collected and annotated to train the detection model.

Establishing a deep-learning-based wild bird detection model requires the completion of two essential tasks. First, a large number of wild bird images must be collected to train the detection model. In this study, for efficiently and effectively collecting wild bird images, a camera with a motion detection function (SNC-VM772R, Sony, Tokyo, Japan) was used. The camera was installed at a farm to capture images of wild birds. Whenever a moving object entered the camera’s field of view, it captured an image with a resolution of 1920 × 1080 pixels. The captured images were manually selected without repetition for deep learning. The criteria for selecting images were those containing wild birds wandering on the ground. Second, the images of wild birds must be annotated for use in deep learning. In this study, VGG Image Annotator software^33^ was used to annotate wild birds in a polygonal format in the collected images.

In this study, to train and validate a wild bird detection model by using mask R-CNN and to evaluate the performance of the trained model, a wild bird image dataset was collected. As displayed in Fig. S1, the images in this dataset were captured from the roof of the agricultural machinery factory at National Chung Hsing University in Taichung, Taiwan; a goose farm in Yunlin, Taiwan; and a duck farm in Yunlin. These fields are dominated by sparrows, mynas, Chinese bulbuls, turtle doves, and pigeons. These wild birds are primarily spotted during the day. Table 1 presents the composition of the dataset used to train the adopted wild bird detection model. The collected dataset was divided into three sections: a training section, validation section, and test section. Table S1 shows wild bird population distribution of each dataset. Most images contain multiple targets. In the training dataset, the total number of target wild bird is 6008, the average number of pixels of the target wild bird is 178, and the standard deviation is 211. In the validation dataset, the total number of target wild bird is 2631, the average number of pixels of the target wild bird is 176, and the standard deviation is 186. In the test dataset, the total number of target wild bird is 1851, the average number of pixels of the target wild bird is 157, and the standard deviation is 135.

**Table 1:** Composition of the image dataset used for training the adopted wild bird detection model.

Dataset part	Number of images	Agricultural machinery factory	Goose farm	Duck farm
Training	320	50	100	170
Validation	250	40	80	130
Test	80	0	0	80

### Model training process and optimization for wild bird detection

In this study, a model trained using the COCO 2017 train/val dataset^34^, named COCO pre-trained model, was selected as a pre-trained model for further training. The COCO 2017 train/val dataset consists of 123,287 images, including 3,362 images with birds. COCO pre-trained model can detect 80 categories of objects, including bird. In our wild bird detection model, only bird object is included. In mask R-CNN, three training parameters are used as adjustable parameters, namely the learning rate, epoch, and anchor scale. In this study, the learning rate was set as 0.001 during the training process because the pre-trained model was fully trained. Thus, a low learning rate was set to achieve a continuously decreasing loss function value. To increase the diversity of the training data, 50% of the training data were randomly flipped horizontally during the mask R-CNN training process in each epoch. An epoch of 300 was selected because the collected wild bird image dataset was relatively small, and additional training and validation epochs were required to achieve improved detection performance. Finally, the anchor scale was set as 8, 16, 32, 64, and 128, which is four times smaller than that used in the literature^30^, because the detection target in this study (i.e., wild birds) occupied a considerably small part of the collected images.

### Evaluation of wild bird detection model

After the mask R-CNN training and validation process, the wild bird detection ability of the optimal trained model, named optimized wild bird detection model, is evaluated using test dataset. To evaluate the trained model, two crucial parameters must be determined: minimum detection confidence and threshold intersection over union (IoU). When a mask R-CNN model is used for target detection, a confidence score is assigned to each target. This confidence score ranges from 0 to 1. When the confidence score increases, the mask R-CNN model becomes more confident that the detected target is correct. After the minimum detection confidence is set, the mask R-CNN detection model can only suggest targets with a confidence score greater than the set minimum detection confidence. To prevent misdetection, the minimum detection confidence is typically set between 0.900 and 0.999. If the detected IoU exceeds the predefined threshold, the predicted target is regarded as a success and is classified as a true positive (TP). By contrast, if the IoU is less than the predefined threshold, the predicted target is regarded as a failure and is classified as a false positive (FP). If the image contains a wild bird that remains undetected, the predicted target is classified as a false negative (FN). After the prediction results are classified, two indicators can be used to quantify the wild bird detection results. The first indicator is precision, which can be defined as Eq. (1). Precision is the ratio between the number of wild birds detected by the trained model and the actual number of wild birds in the image. The second indicator is recall, which can be defined as Eq. (2). Recall is the proportion of wild birds successfully detected by the trained model relative to the total number of wild birds in the image. For the object detection model, greater precision and recall indicate that the trained model is more capable of detecting wild birds. When the wild bird detection model was integrated within the laser repellent system, the laser repel system could only project one laser to repel wild birds during each repel operation. In addition, most wild birds usually move in flocks. When the system detects a single wild bird within the flock, the laser could be activated for repelling them. Therefore, in automatic wild bird repellent systems, higher precision holds greater significance than higher recall. The greater precision allows the system to repel wild birds by laser more effectively and specifically.

1 $$\text{Precision}=\frac{TP}{TP+FP}$$

2 $$\text{Recall}=\frac{TP}{TP+FN}$$

As presented in Table 2, to evaluate the trained wild bird detection model, different minimum detection confidence and threshold IoU values were used to calculate the corresponding precision and recall. The minimum detection confidence was set between 0.90 and 0.95, and the threshold IoU was set between 0.5 and 0.1. In object-detection-related research, the threshold IoU is most commonly set as 0.5. In this study, a threshold IoU of 0.1 was selected to represent the detection of the birds’ surroundings. This setting allowed the proposed system to project a laser beam around wild birds to repel them, thereby fulfilling the requirement of this study. Further analysis was conducted on the setting with the highest precision, and the test dataset was divided into two parts based on the average pixels of ground truth wild bird annotations, and the impact of larger and smaller pixels of wild birds on the detection effect was analyzed.

**Table 2:** Parameters of the wild bird detection experiment.

Setting	Detection minimal confidence	Threshold IoU
I	0.90	0.5
II	0.90	0.1
III	0.95	0.5
IV	0.95	0.1

### Automatic wild bird repellent system

**Figure 2:**
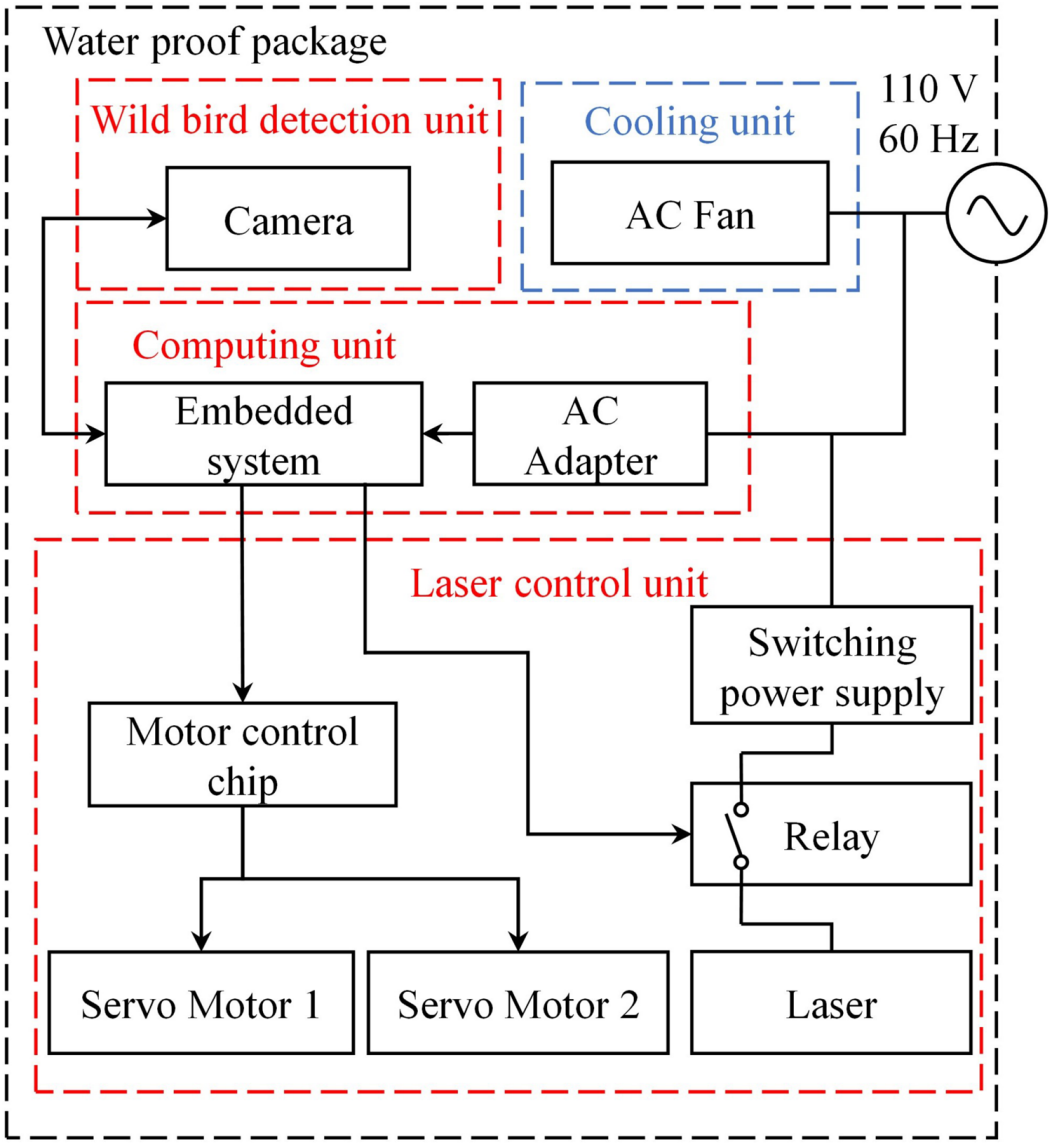
Architecture of the proposed automatic wild bird repellent system.

Figure 2 depicts the architecture of the proposed automatic wild bird repellent system. This system consists of three units: a wild bird detection unit, computing unit, and laser control unit. The wild bird detection unit contains a C922 Pro Stream camera (Logitech, Lausanne, Switzerland) that captures images of the relevant farm and delivers them to the computing unit. The computing unit contains a Jetson TX2 embedded system (Nvidia, Santa Clara, CA, USA) that runs the adopted wild bird detection model trained through mask R-CNN deep learning. The operating system of the proposed wild bird repellent system’s computing unit, Jetson TX2, is Ubuntu18.04. The programming language for executing mask R-CNN and communicating with other hardware is python. The embedded system is connected to a relay and motor control chip (PCA9685; Adafruit Industries, New York, NY, USA) in the laser control unit. The a-contact of the relay is connected to a 400-mW green laser source with a wavelength of 505–530 nm (VLM-520; Quarton, Taipei City, Taiwan). This laser can be switched on and off by the embedded system’s signal-controlled relay. The motor control chip is connected to two servo motors (SA-1256TG and SC-1251MG; SAVOX, Taichung City, Taiwan). The laser is mounted on these two motors to control the direction of the projected laser light. Because the proposed system is designed to operate in outdoor environments, it requires a waterproof enclosure and cooling unit. Therefore, an alternating-current (AC) fan (GA2082HSL-A, Gulf Electrics, Kaohsiung, Taiwan) is used to decrease the internal temperature of the proposed system through heat exchange. An external AC 110-V power supply is used to provide the required power through an AC adapter to the embedded system, and a switching power supply (RS-15-5; MEAN WELL, New Taipei City, Taiwan) is used to power the laser source.

**Figure 3:**
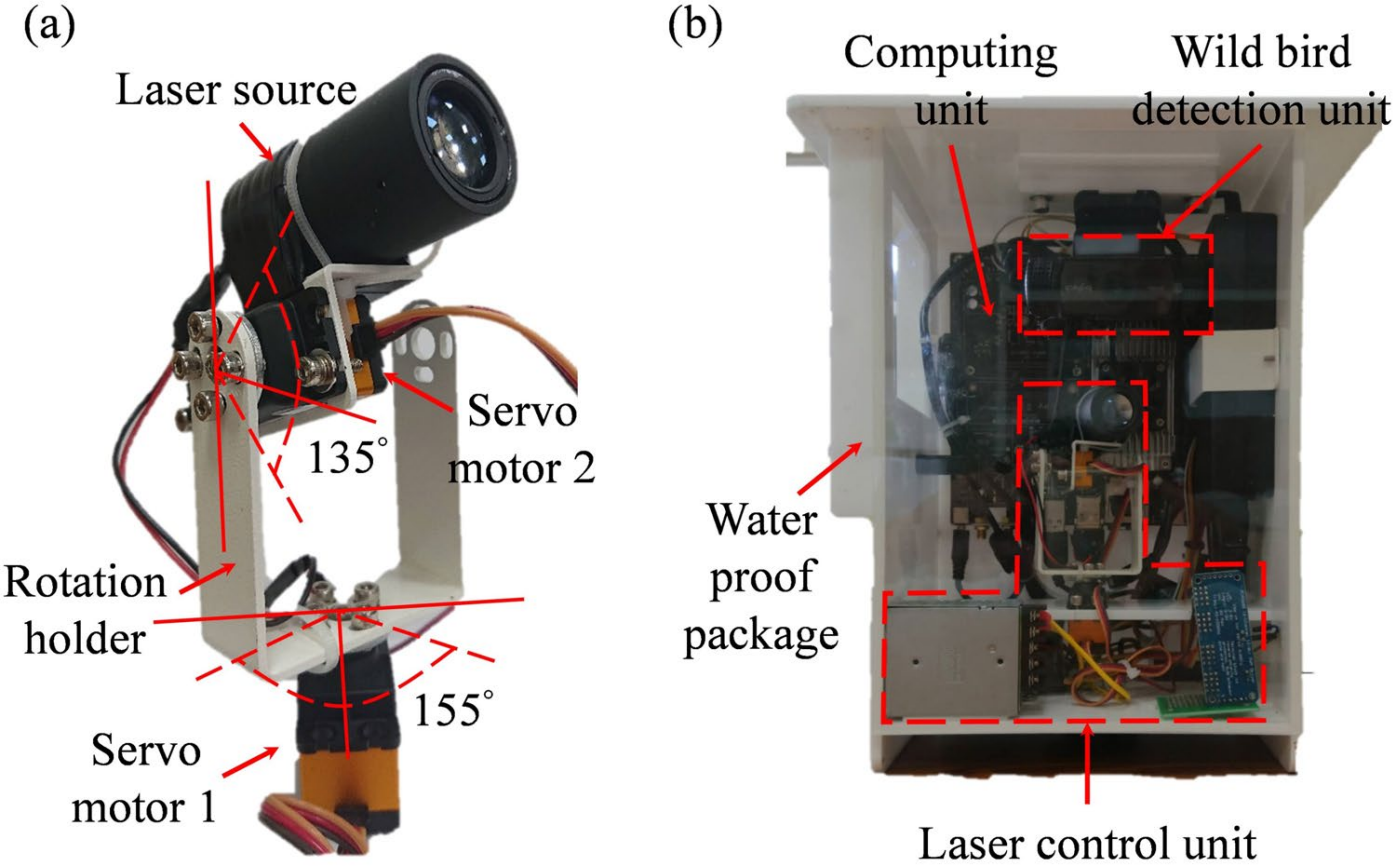
Images of the (**a**) laser rotation mechanism and (**b**) proposed automatic wild bird repellent system.

As shown in Fig. 3a, the laser rotation mechanism consists of two servo motors, which are used to control the direction of the laser. The first servo motor has a large torque and is fixed at the bottom of the laser rotation mechanism to achieve horizontal rotation in the rotation holder. The maximum programmable control angle for the first servo motor is 155°. The second servo motor is fixed between the rotation holder and the laser source to achieve the vertical rotation of the laser light. The maximum programmable control angle for the second servo motor is 135°. Figure 3b depicts the proposed automatic wild bird repellent system. This system has a length, width, and height of 27, 20, and 30 cm, respectively, and it can be used in poultry farms by connecting it to a 110-V, 60-Hz AC power supply. The system is also waterproof and can thus be used outdoors.

**Figure 4:**
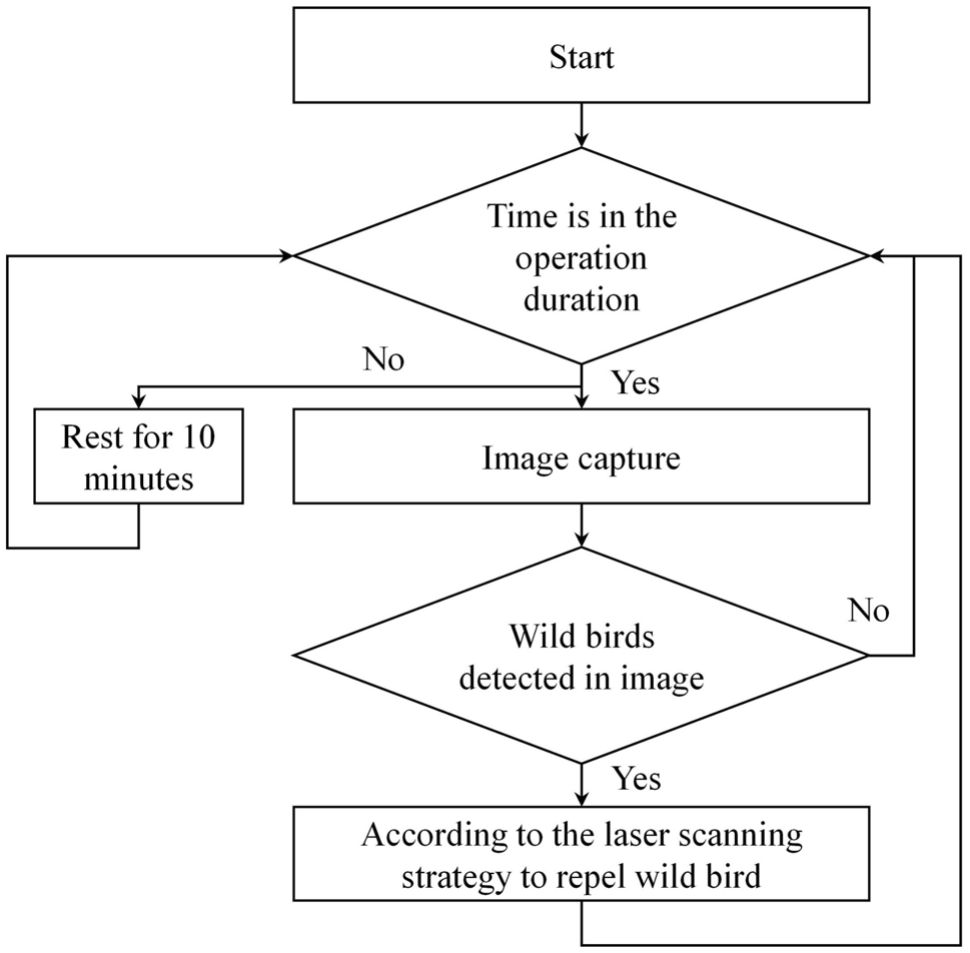
Operational process flow of the proposed automatic wild bird repellent system.

Figure 4 depicts the operational process flow of the proposed automatic wild bird repellent system. If the current time falls within the operation period, the embedded system activates the camera and captures an image at a resolution of 1920 × 1080 pixels. The trained mask R-CNN model then detects wild birds in the captured image. If no wild birds are detected, the process of confirming the current time is resumed. However, if wild birds are detected, the embedded system controls the relay to activate the laser. Simultaneously, the embedded system controls the two servo motors to project laser light around the detected wild birds to repel them. Following this process, the motors return to their initial state, and the system resumes the process of confirming the current time. If the current time falls within the nonoperation period, the system rests for 10 min.

### Laser scanning strategies

Most wild bird repellent method by lasers is carried out manually by manpower or through periodic laser scanning within a predefined range, following programmed waypoints and schedules^9,21^. The wild bird repellent system proposed in this study could detect wild birds firstly. The important elements that affect the bird repelling effect of the wild bird repelling system are the wild bird detection model and the laser scanning strategy. The model with better detection ability would enhance the bird repelling ability of the system. Then the laser could accurately project around the wild birds for enhancing the precision of laser bird repelling task. Recent literature has not discussed whether different laser scanning strategies will affect the bird repellent effect. To effectively repel wild birds by laser, it is crucial to design an appropriate laser scanning region and scanning path.

**Figure 5:**
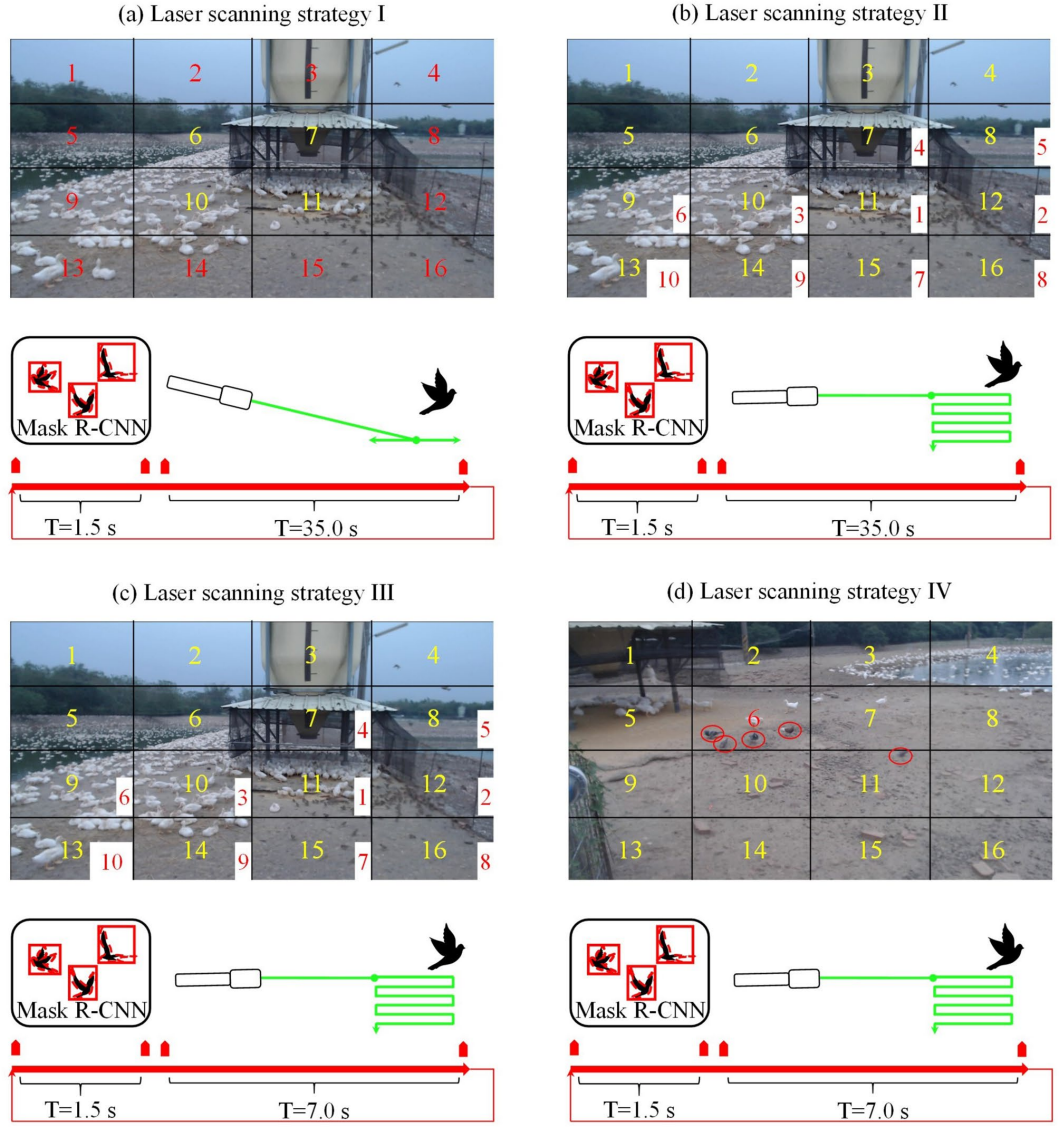
Illustration of the four laser scanning strategies adopted in this study.

In this study, four laser scanning strategies were evaluated. As displayed in Fig. S2, the captured image was divided into 16 regions. After the mask R-CNN wild bird detection process, the wild bird positions in the captured image were determined. This step enabled the detection of the number of wild birds in each region. The four adopted laser scanning strategies are illustrated in Fig. 5 and described in Table 3.

**Table 3:** Information on the four laser scanning strategies adopted in this study.

Strategy number	Priority regions for laser repelling	Laser scanning path	Laser scanning angle	System execution cycle time (s)
I	Center regions of the captured image	Horizontal line scanning	Horizontal 10°	36.5
II	Center regions of the feed area	Horizontal and vertical line scanning	Horizontal 10° Vertical 6°	36.5
III	Center regions of the feed area	Horizontal and vertical line scanning	Horizontal 10° Vertical 6°	8.5
IV	Region with the greatest number of wild birds	Horizontal and vertical line scanning	Horizontal 10° Vertical 6°	8.5

In laser scanning strategy I, the priority regions are the four central regions of the image. When wild birds are detected in any of these regions, a random region is selected for laser scanning. If no wild birds are detected in the four central regions but some are detected in the peripheral regions, one of the peripheral regions is randomly selected for laser scanning. As shown in Fig. 5a, the laser is projected horizontally at an angle of 10° for 35 s. Laser scanning strategy I has an execution time of approximately 36.5 s, which includes the time required for the mask R-CNN detection process. In laser scanning strategy II, the laser scanning priority region is determined by the distance to the feed area, as depicted in Fig. 5b. The scanning process is performed in the following order: region 11 → region 12 → region 10 → region 7 → region 8 → region 9 → region 15 → region 16 → region 14 → region 13. If wild birds are detected in these regions, the first region containing wild birds is selected for laser scanning. If wild birds are detected in other regions, the laser is not activated. In the aforementioned strategy, the laser is projected horizontally at an angle of 10° and vertically at an angle of 6°. Laser scanning strategy II has an execution time of approximately 36.5 s, and its scanning region priority and laser scanning path differ from those of laser scanning strategy I. In laser scanning strategy III, the scanning region priority and laser scanning path are identical to those of laser scanning strategy II, as displayed in Fig. 5c. The difference between these two strategies is related to their laser scanning times. The laser scanning speed in laser scanning strategy III is higher than that in laser scanning strategy I; thus, the laser scanning time is reduced from 35 s in laser scanning strategy I to 7 s in laser scanning strategy III, which results in an increased system detection and repulsion frequency in laser scanning strategy III. In laser scanning strategy IV, the priority region is the region with the largest number of detected wild birds, as depicted in Fig. 5d. In this laser scanning strategy, the laser scanning path is identical to that of laser scanning strategy III. The execution time of laser scanning strategy IV is approximately 8.5 s.

### Field experiments and setup

The proposed automatic wild bird repellent system was installed and tested at a duck farm in Yunlin, Taiwan. Fig. S3 depicts the general layout of the duck farm. The farm has a semiopen duckling care cabin and five outdoor feeding areas and is surrounded by agricultural land, orchards, and woods. Therefore, various wild birds visit the farm during the day to feed. According to the farm owner and the image data collected on site, sparrows, mynas, Chinese bulbuls, turtle doves, and pigeons are the predominant species of wild birds spotted at the farm. The most frequent locations at which these wild birds are spotted are around the feed buckets. However, at nighttime, no wild birds are spotted in the field.

**Figure 6:**
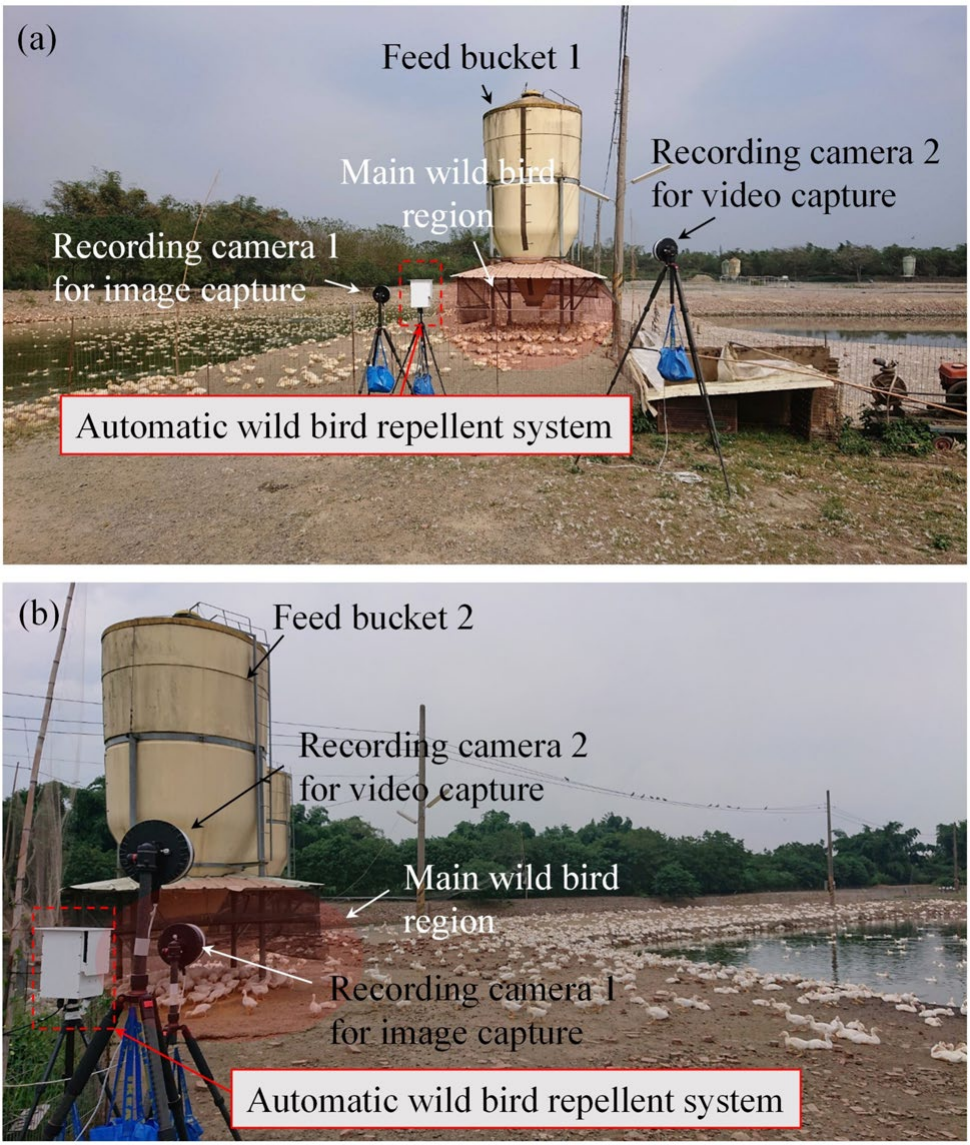
Experimental field setups for (**a**) feed bucket 1 and (**b**) feed bucket 2.

**Figure 7:**
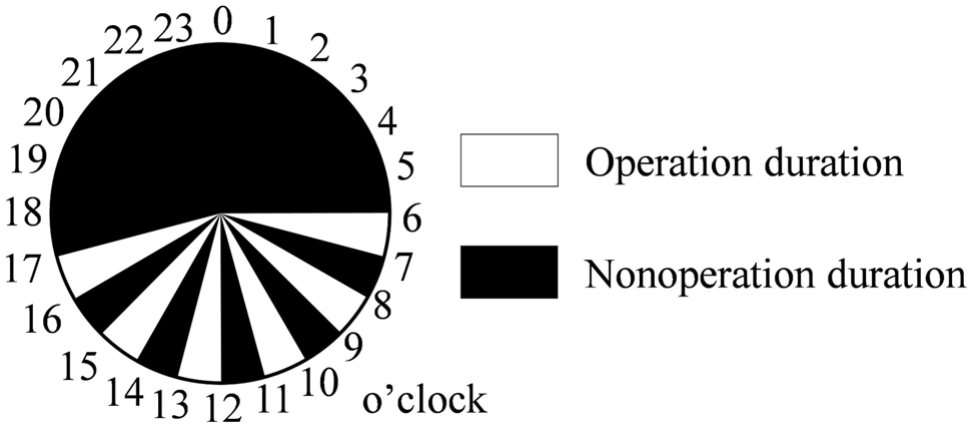
Duration of operation of the proposed automatic wild bird repellent system during the field experiments.

Figure 6a and b depict the experimental setups of feed buckets 1 and 2, respectively. The proposed automatic wild bird repellent system was installed on a tripod and oriented toward the feed buckets. To prevent the system from collapsing as a result of a wind gust or duck collision, the tripod was weighted with heavy objects. Two cameras were installed adjacent to the proposed system to record the experimental process. Recording camera 1 was used to capture an image at a resolution of 1920 × 1080 pixels every minute for quantifying the experimental results. Recording camera 2 was used to capture a video at a resolution of 1920 × 1080 pixels and a frame rate of 10 fps to observe the experimental process. Figure 7 depicts the duration of operation of the automatic wild bird repellent system. Because the majority of wild birds are active during the day, the experiments were conducted during the day. To determine the effectiveness of the proposed system, the system was alternately operated for 1 h and then switched off for 1 h. Four experiments were conducted, each of which was performed over 4 consecutive days, as presented in Table 4. In the four experiments, the aforementioned four laser scanning strategies were used and compared. To prevent wild birds from becoming accustomed to the laser, the interval between each experiment was set as at least 1 week.

**Table 4:** Detailed information on the field experiments conducted at the duck farm.

Experiment	Date of experiment	Strategy number	Target area	Ducks in target area
I	March 10th, 2020 to March 14th, 2020	I	Feed bucket 1	Yes
II	April 17th, 2020 to April 21st, 2020	II	Feed bucket 1	Yes
III	May 29th, 2020 to June 2nd, 2020	III	Feed bucket 1	No
IV	June 10th, 2020 to June 14th, 2020	IV	Feed bucket 2	Yes

### Method of quantifying the experimental results

Most studies on wild bird repellents have been based on subjective human assessments of the effectiveness of bird repellent methods. The number of wild birds in the field was estimated by the density of wild bird droppings^9^. The number of wild birds was counted by experienced ornithologists watching field video recording, and statistics were made according to different species of wild birds^21^. However, the method of manually counting the number of wild birds is very labor-intensive, time consuming, and not suitable for fields with a lot of wild birds. In this study, a rapid and objective method was proposed for quantifying the results of bird repellent experiments. Recording camera 1 was used to capture an image every minute. After each experiment, the same trained mask R-CNN model was used to determine the number of wild birds in each image captured by recording camera 1. To estimate the number of wild birds at a field during a given period, the number of wild birds detected in each image captured during this period was summed. This objective method can be used to quantify a large number of experimental results. In addition, determining the number of wild birds in an image by using a mask R-CNN model can shorten the duration of experimental data analyses and reduce labor requirements. In this study, two indicators were used to determine the effectiveness of automatic bird repelling. The first indicator was daily bird repulsion rate (*BRR*_*d*_), which is defined as Eq. (3), where *N*_on_ and *N*_off_ are the numbers of wild birds in each image captured in 1 h with the proposed automatic wild bird repellent system switched on and off, respectively, and the subscript *i* indicates a distinct hour. The sum of the number of wild birds can be used to estimate the scenario of wild bird appearance at the field. In addition, the difference in the number of wild birds between the system’s active and inactive states can be used to estimate the repulsive effect of the system. To achieve a fair comparison, the same calculation method should be used for determining the number of wild birds per unit time. According to the experimental design, images captured from 06:00 to 18:00 on a single day were used for calculations. Thus, the system was switched on for 6 h and switched off for 6 h. The effectiveness of the proposed wild bird repellent system was determined by comparing the numbers of wild birds at the field when the system was switched on and off on a single day. Therefore, in addition to the repellent effect in 1 day, the repellent effect in 1 h was estimated. The second indicator used to estimate the bird repellent effect of the proposed system was hourly bird repulsion rate (*BRR*_*h*_), which is defined Eq. (4). To estimate the wild bird repellent effect in a single hour, the number of wild birds during 1 h of activity (*N*_on*,i*_) was compared with the total number of wild birds before and after the operation period. The parameter *BRR*_*h*_ was used to monitor the effectiveness of the proposed wild bird repellent system in 1 h of operation.

3 $${BRR}_{d}=\frac{\sum_{In\, one\, day}{N}_{off,i+1}-\sum_{In \,one\, day}{N}_{on,i}}{\sum_{In\, one\, day}{N}_{off,i+1}}\times 100\%$$

4 $${BRR}_{h}=\frac{\frac{({N}_{off,i-1}+{N}_{off,i+1})}{2}-{N}_{on,i}}{\frac{({N}_{off,i-1}+{N}_{off,i+1})}{2}}\times 100\%$$

### Statistical analysis

Statistical analysis was performed for the four field experimental results, and the minimum sample size required for the statistical test was calculated. To assess repel efficacy of the proposed wild bird repellent system, the wild bird number observed per hour was divided into two groups. These two groups are wild bird numbers with system turned-on as treatment group and wild bird numbers with system turned-off as control group. To test whether the system has a significant effect on wild bird number, a negative binomial regression model was fitted, which corrects for overdispersion observed when fitting a Poisson regression model, using the library MASS in the statistical software package R version 4.3.0.

## Results

### Evaluation of wild bird detection

Table 5 shows evaluation results of the adopted wild bird detection model under four setting of minimum detection confidence and threshold IoU values. The highest precision 0.865 was obtained when using setting IV under a minimum detection confidence of 0.95 and a threshold IoU of 0.1. Therefore, the setting IV was adopted for the proposed wild bird repellent system. Fig. S4 shows visual examples of the wild bird detection results at the feed bucket 1 and the feed bucket 2. Fig. S4a and b are original images captured from the camera. Fig. S4c and d show detection results with the COCO pre-trained model. Fig. S4e and f show detection results with the optimized wild bird detection model. Table 6 shows evaluation results of the optimized wild bird detection model under different target pixel values in test dataset. Fig. S5 shows visual examples of the false detection of wild birds. Subsequently, calculate the ratio of the number of falsely detected ducks by the optimized wild bird detection model to the manually counted number of ducks in the image. The ratio in Fig. S5(a) is 2/150, and the ratio in Fig. S5(b) is 1/55.

**Table 5:** Evaluation results of the adopted wild bird detection model under four setting of minimum detection confidence and threshold IoU values.

Setting	Precision & Recall with COCO pre-trained model	Precision & Recall with optimized wild bird detection model
I	0.001 & 0.001	0.607 & 0.100
II	0.001 & 0.001	0.793 & 0.131
III	0.000 & 0.000	0.743 & 0.059
IV	0.000 & 0.000	0.865 & 0.069

**Table 6:** Evaluation results of the optimized wild bird detection model under different target pixel values in test dataset.

Target size (pixels)	Number of targets	Number of detected targets	Detection percentage
≤ 157	1227	30	2.44%
> 157	624	98	15.71%

### Experimental field results for the repelling of wild birds

**Figure 8:**
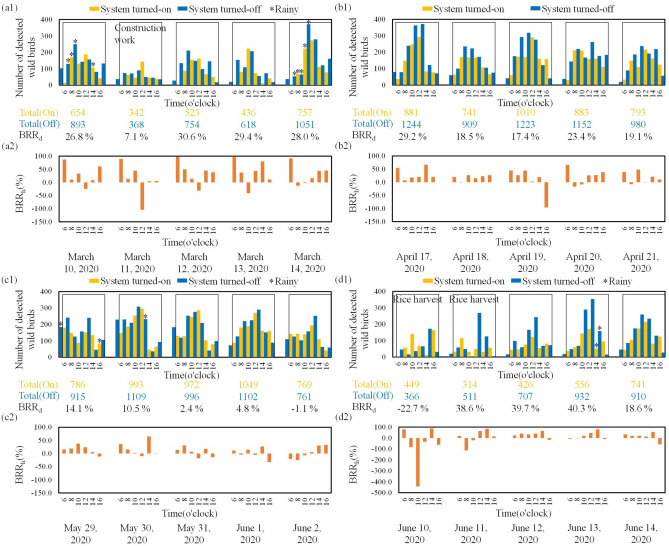
Bird repulsion results: (**a1**) number of wild birds every hour and daily wild bird repulsion rate and (**a2**) calculated hourly wild bird repulsion rate for field experiment I. (**b1**) number of wild birds every hour and daily wild bird repulsion rate and (**b2**) calculated hourly wild bird repulsion rate for field experiment II. (**c1**) number of wild birds every hour and daily wild bird repulsion rate and (**c2**) calculated hourly wild bird repulsion rate for field experiment III. (**d1**) number of wild birds every hour and daily wild bird repulsion rate and (**d2**) calculated hourly wild bird repulsion rate for field experiment IV.

Figure 8 depicts the number of detected wild birds, and calculated daily and hourly bird repulsion rates in each experiment. The numbers of wild birds detected with the proposed system switched on and off were recorded. The daily bird repulsion rate (*BRR*_*d*_) was calculated from 06:00 to 18:00. Thus, the system was switched on and switched off for 6 h. Table 7 shows the *BRR*_*d*_ and *BRR*_*h*_ values for each experiment. As shown in Fig. 8(a1), light rains were observed on the first and fifth mornings, and construction work was observed on the second day. As shown in Fig. 8(b1), no precipitation was observed. As shown in Fig. 8(c1), heavy precipitation was observed on the first day's afternoon. Because of this heavy precipitation, the number of wild birds decreased on the first day. As shown in Fig. 8(d1), heavy precipitation was observed on the afternoon of the fourth day, and the first two days of the experiment coincided with the nearby rice harvest, which resulted in a considerable reduction in the total number of wild birds in the field.

**Table 7:** *BRR*_*d*_ and *BRR*_*h*_ values for each experiment.

Experiment	Day	BRR_d_ (%)	BRR_h_ (%)					
			6	8	10	12	14	16
I	1	26.8	87.1	10.5	33.3	− 24.7	8.4	60.6
	2	7.1	89.2	13.7	44.4	− 104.3	4.2	4.9
	3	30.6	97.5	49.6	13.6	− 31.7	44.9	38.7
	4	29.4	96.6	37.6	− 41.1	43.7	79.8	10.9
	5	28.0	91.2	− 12.6	− 2.3	16.0	44.0	44.7
II	1	29.2	54.1	7.3	17.9	20.4	66.7	20.2
	2	18.5	20.0	− 0.9	26.6	15.2	22.5	26.7
	3	17.4	44.7	26.5	44.3	2.4	19.4	− 96.3
	4	23.4	65.6	− 16.8	− 8.5	25.9	27.1	38.3
	5	19.1	38.9	− 7.6	48.0	− 0.7	21.2	10.1
III	1	14.1	15.3	19.3	37.8	23.9	5.3	− 11.3
	2	10.5	35.9	15.5	2.3	− 9.6	64.9	0.8
	3	2.4	13.9	31.2	7.0	− 17.9	17.6	− 13.7
	4	4.8	11.6	− 4.3	14.6	− 4.9	27.0	− 32.5
	5	− 1.1	− 20.3	− 24.9	− 6.5	5.4	30.2	32.8
IV	1	− 22.7	80.0	− 83.9	− 442.3	− 34.7	88.2	− 61.6
	2	38.6	17.9	− 113.1	− 20.8	64.1	84.3	14.5
	3	39.7	22.8	40.5	32.7	39.9	64.6	− 15.7
	4	40.3	− 7.5	− 2.6	19.9	46.7	80.0	− 9.8
	5	18.6	33.3	19.2	20.1	13.2	55.5	− 58.0

### Wild bird repellent efficacy of proposed system

According to the results of the four field experimental results, the average number of wild birds with system turned-off is 146 with standard deviation of 90, and the average number of wild birds with system turned-on is 117 with standard deviation of 73. Under 95% confidence level and 90% statistical power, and considering the above-mentioned statistical results, the calculated minimum sample size required to test the bird repelling effect of the system is 83 h with the system turned on and 83 h without the system turned off. The results of analyzing the experimental data using negative binomial regression are shown in Table 8.

**Table 8:** Output from the negative binomial regression model describing system performance with and without environmental interference.

Environmental disturbance	On/off sample size	Effect	*p* value	Estimate
Yes	120/120	Total_Intercept		4.983
		Total_System On/Off	0.017	− 0.218
No	95/96	Total_Intercept		5.062
		Total_System On/Off	0.022	− 0.211

Both *p* values indicate significant effects on α-level 0.05.

## Discussion

When using the COCO pre-trained model for evaluation, it can be found from the results that no matter what setting is used, the performance of detecting small target wild birds is very poor. Using the optimized wild bird detection model for evaluation, it can be found from the results that no matter what setting is used, the performance of detecting small target wild birds is better than the former one. Compared to the COCO pre-trained model, the small bird target detection performance of the optimized wild bird detection model is significantly improved. Although this study has improved the detection ability of small target wild birds, further analysis shows that the detection performance of wild birds below the average pixels is still relatively poor compared to the detection performance of wild birds above the average pixels. This issue would be improved in the future. Observing the visual examples of the false detection of wild birds, even if the image is full of ducks, the optimized wild bird detection model still has a low ratio of falsely detecting ducks as wild birds.

A high precision value indicated that a large proportion of detected wild birds were correct by the trained model, which suggested that laser light was successfully projected around the wild birds to repel them. By contrast, a low recall value indicated that the trained model could not detect many wild birds in the image because these birds occupied a small number of pixels in the captured image. A low recall value does not considerably affect the proposed wild bird repellent system because this system operates continuously as long as a bird is detected in the captured image.

In field experiment I, the total number of wild birds and the *BRR*_*d*_ value on day 2 were considerably lower than those on the other days because some construction work was being conducted at the duck farm on day 2. The movement of and noise from the construction vehicles at the farm resulted in a decline in the number of wild birds, which caused a reduction in the *BRR*_*d*_ value. According to the collected data, the number of wild birds decreased during the periods of precipitation. The highest *BRR*_*h*_ value was observed at 06:00 each day, which indicated that the proposed system effectively repelled wild birds. However, at 12:00, the *BRR*_*h*_ value decreased or even became negative because the ambient light was strong around noon, which made it difficult for wild birds to see the laser light. Therefore, the efficacy of wild bird repulsion decreased around noon.

In field experiment II, the *BRR*_*h*_ values on the 5 days were predominantly positive, and the proposed system repelled wild birds effectively. However, some negative *BRR*_*h*_ values were observed, which indicated that the effectiveness of wild bird repulsion was negligible or nonexistent. These values were obtained because the ambient light was too bright, and the laser light was not sufficiently visible to the birds to repel them. The lowest *BRR*_*h*_ value of − 96.3% was recorded at 16:00 on day 3 of the experiment. To understand the ground situation, a video was recorded. According to this video, at 16:00 on day 3, when laser light was projected around the wild birds, some of them did not fly away from the laser scanning area. Consequently, the *BRR*_*h*_ value calculated from the captured images was quite low during this hour.

In field experiment III, the results indicated that *BRR*_*d*_ tended to decrease gradually as the number of experimental days increased. Analysis of the recorded video indicated that the birds might have become accustomed to the laser scanning path, which was also observed in field experiment II. Although the *BRR*_*h*_ values were predominantly positive, negative values began to appear irregularly.

In field experiment IV, the highest *BRR*_*d*_ value was observed on the fourth day, which indicated that the proposed system repelled wild birds effectively. On the first day, the *BRR*_*d*_ value was − 442.3% at 10:00. According to the recorded video, the birds became accustomed to the laser scanning path and were not affected by the laser, which was also observed in field experiments II and III. The aforementioned results can be attributed to the fact that when wild birds are repelled by the same laser scanning path for an extended period, they lose their fear of the laser. In addition, ambient light plays a critical role in the laser repulsion effect. At 10:00 on day 1, the ambient light was excessively bright, which made it difficult for the wild birds to see the laser light. In field experiment IV, the *BRR*_*h*_ values were predominantly positive, which indicated that the proposed system repelled wild birds effectively.

The highest hourly wild bird repulsion rate was 97.1%, and the highest daily wild bird repulsion rate was 40.3%. However, in certain conditions, such as when the ambient light was too bright, the wild bird repulsion rate temporarily decreased. Therefore, the laser scanning strategy must be enhanced in the future to increase the wild bird repulsion rate.

The statistical test result of all experimental data shows that whether the system is turned-on or turned-off has a significant effect on the number of wild birds observed (*p* < 0.05). After excluding the experimental data of environmental disturbances, such as rainfall, construction work, and rice harvest, the statistical test result shows that whether the system is turned-on or turned-off has a significant effect on the number of wild birds observed (*p* < 0.05). The presence or absence of the system had a significant negative effect on wild bird number, indicating that the system is effective for repelling wild birds.

The limitation of the proposed wild bird repellent system is that wild birds would not see the laser spot when weather is very sunny. The same laser scanning path would cause wild birds habituated and result in decreasing the efficacy of the wild bird repellent system. Therefore, changing the laser scanning path is one way to reduce wild bird to laser scanning path habituation, and increasing the number of laser spots is another effective approach. Future research directions include increasing the mechanism of rotating the entire bird repellent system for wider repelling work, increasing the speed of laser repelling, implementing different repelling strategies based on different species of birds. Furthermore, for improving the performance of small target detection in birds, super-resolution assisted methods such as SuperYOLO^35^ or YOLOX-Small^36^ could be implemented. For addressing misclassification issues in object detection and instance segmentation algorithms, the latest YOLOv7^37^ could be implemented, and it would be beneficial to consider advanced pose estimation algorithms^38^. For identifying wild birds, incorporating multi-camera systems or multimodal data analysis might be considered^35^. If birds in flight need to be repelled in the future, tracking algorithms will have to be introduced. For epidemic prevention, although the reduction in the wild bird number could intuitively reduce the risk of disease transmission, further experiments still need to be carried out.

## Conclusion

In this work, an automatic wild bird repellent system based on deep-learning-based wild bird detection and integrated with a laser rotation mechanism was proposed and developed. The wild bird detection model of the proposed system was optimized for detecting small pixel targets, and trained through a deep learning method by using wild bird images captured at different farms. Compared to the COCO pre-trained model, the small bird target detection performance of the optimized wild bird detection model is significantly improved. A rapid and objective method was proposed for quantifying the results of bird repellent experiments. This method could be used to quantify a large number of experimental results. Two indicators were used to determine the effectiveness of automatic bird repelling. The first indicator was daily bird repulsion rate (*BRR*_*d*_), and the second indicator was hourly bird repulsion rate (*BRR*_*h*_). Four wild bird repulsion experiments were conducted using the proposed system at an outdoor duck farm. The statistical test results of our experimental data indicated that the proposed automatic wild bird repellent system effectively reduced the number of wild birds in the farm. The experimental results indicated that the developed system effectively repelled wild birds, with a high repulsion rate of 40.3% each day.

### Supplementary Information

Supplementary Information 1.

Supplementary Information 2.

## Data Availability

Data is provided within the manuscript or supplementary information files.
