# Supplementary material for: Automatic wild bird repellent system that is based on deep-learning-based wild bird detection and integrated with a laser rotation mechanism
Source: Sci Rep. 2024 Jul 10;14:15924. doi: 10.1038/s41598-024-66920-2 (PMC11237150; doi:10.1038/s41598-024-66920-2)
Supplement: Supplementary file 1 — Supplementary Information 1. [file 41598_2024_66920_MOESM1_ESM.docx]

Automatic wild bird repellent system that is based on deep-learning-based wild bird detection and integrated with a laser rotation mechanism

Yu-Chieh Chen^1^, Jing-Fang Chu^2^, Kuang-Wen Hsieh^2,3^, Tzung-Han Lin^4^, Pei-Zen Chang^1^, and Yao-Chuan Tsai^2,3,*^

^1^ National Taiwan University, Institute of Applied Mechanics, Taipei, 106319, Taiwan
^2^ National Chung Hsing University, Department of Bio-Industrial Mechatronics Engineering, Taichung, 402202, Taiwan
^3^ Smart Sustainable New Agriculture Research Center (SMARTer), Taichung, 402, Taiwan
^4^ National Taiwan University of Science and Technology, Graduate Institute of Color and Illumination Technology, Taipei, 106335, Taiwan
^*^ yctsaii@dragon.nchu.edu.tw

**
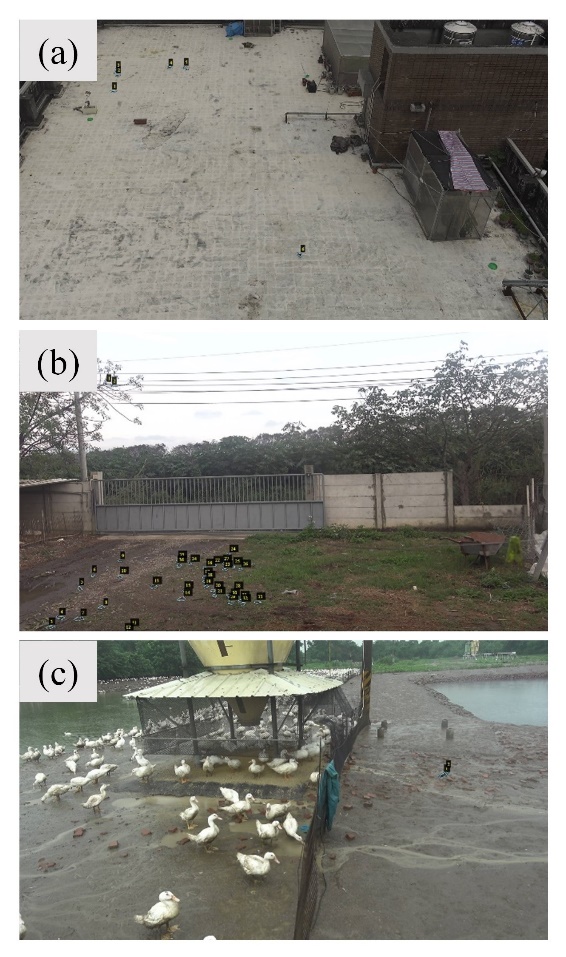
**

**Fig. S1.** Wild bird images captured from (a) the roof of the agricultural machinery factory at National Chung Hsing University in Taichung, (b) a goose farm in Yunlin, and (c) a duck farm in Yunlin.


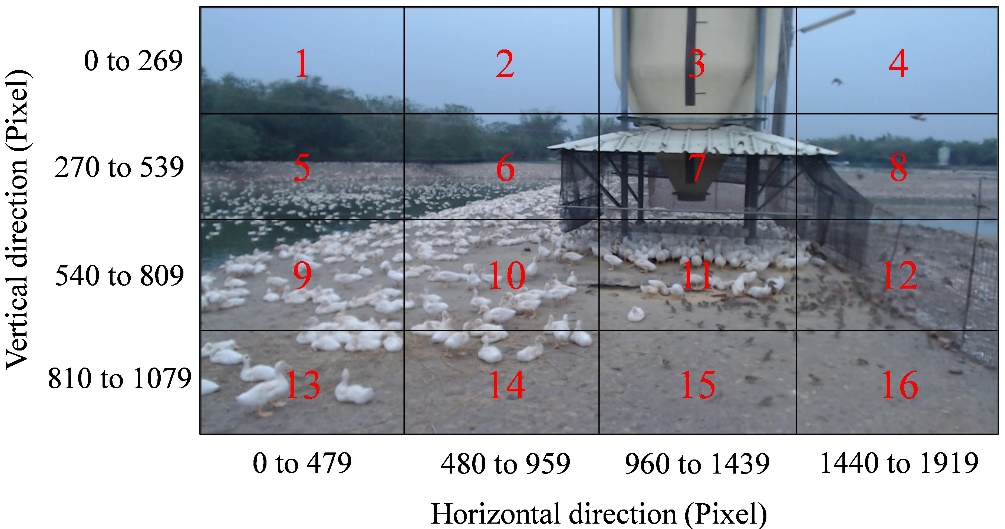


**Fig. S2.** Division of a captured image into 16 regions.


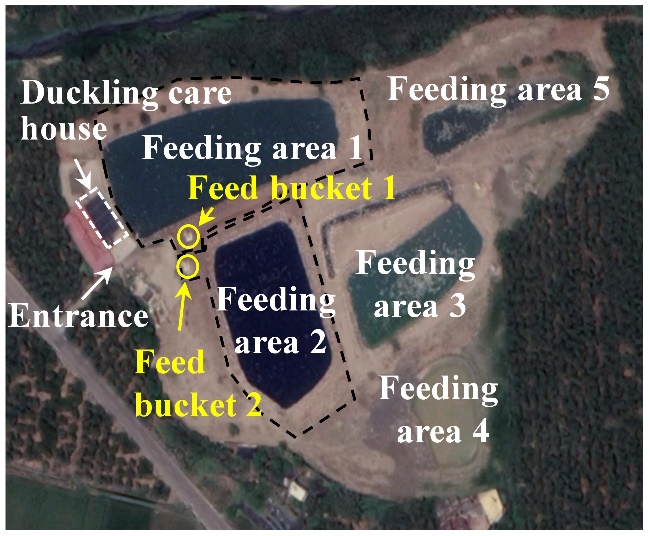


**Fig. S3.** Aerial photo of the experimental field (source: Google Earth, 2020).


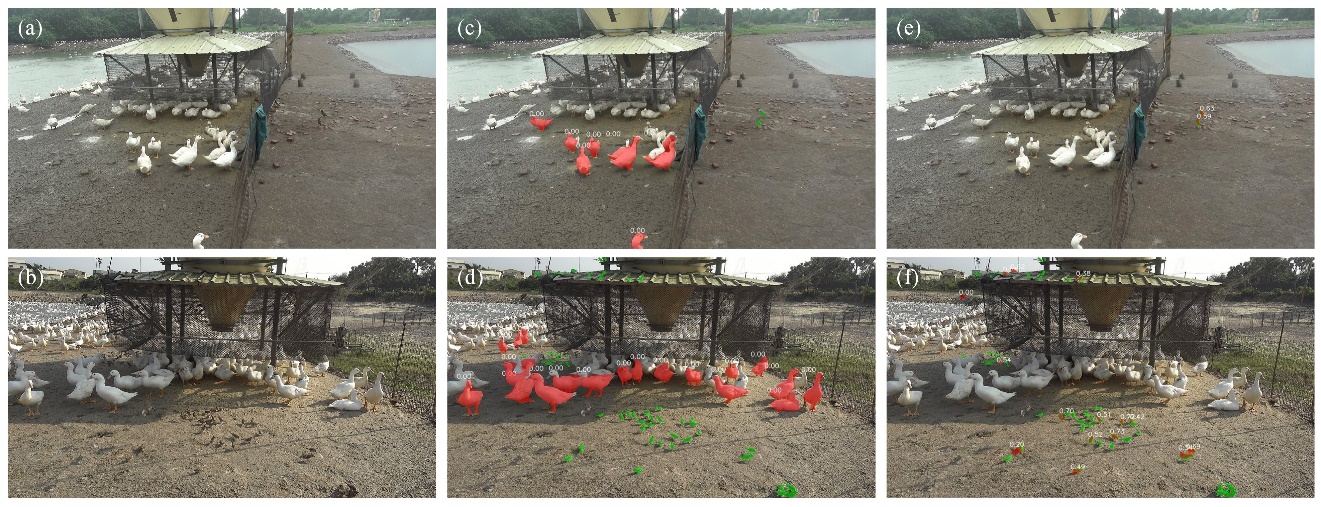


**Fig. S4.** Visual examples of the wild bird detection results at the feed bucket 1 and the feed bucket 2. (a)(b) Original image. (c)(d) Detected with COCO pre-trained model. (e)(f) Detected with optimized wild bird detection model.


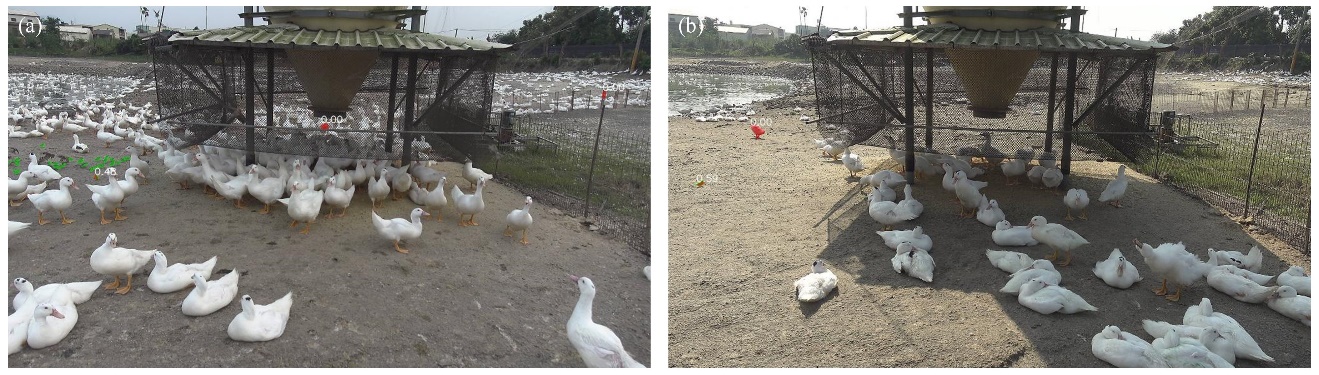


**Fig. S5.** Visual examples of the false detection of wild birds.

**Table S1.** Wild bird population distribution of each dataset.

| **Range of**  **bird number** | **Number of images in**  **the training dataset** | **Number of images in**  **the validation dataset** | **Number of images**  **in the test dataset** |
| --- | --- | --- | --- |
| 1~10 | 179 | 172 | 39 |
| 11~20 | 60 | 41 | 8 |
| 21~30 | 26 | 13 | 7 |
| 31~40 | 12 | 16 | 6 |
| 41~50 | 11 | 3 | 6 |
| 51~60 | 4 | 2 | 6 |
| 61~70 | 8 | 0 | 2 |
| 71~80 | 10 | 2 | 4 |
| 81~90 | 4 | 1 | 2 |
| 91~100 | 2 | 0 | 0 |
| 101~110 | 1 | 0 | 0 |
| 111~120 | 0 | 0 | 0 |
| 121~130 | 0 | 0 | 0 |
| 131~140 | 1 | 0 | 0 |
| 141~150 | 0 | 0 | 0 |
| 151~160 | 0 | 0 | 0 |
| 161~170 | 1 | 0 | 0 |
| 171~180 | 0 | 0 | 0 |
| 181~190 | 1 | 0 | 0 |
